# Luminance white noise electroretinograms (wnERGs) in mice

**DOI:** 10.3389/fnins.2022.1075126

**Published:** 2022-12-09

**Authors:** Nina Stallwitz, Anneka Joachimsthaler, Jan Kremers

**Affiliations:** ^1^Section for Retinal Physiology, Department of Ophthalmology, University Hospital Erlangen, Erlangen, Germany; ^2^Department of Biology, Friedrich-Alexander- Universität Erlangen-Nürnberg, Erlangen, Germany

**Keywords:** electroretinogram (ERG), temporal white noise (TWN), white noise electroretinogram (wnERG), impulse response function (IRF), mice, ERG generating mechanisms, modulation transfer function (MTF)

## Abstract

**Purpose:**

To record and analyse electroretinograms (ERGs) to luminance stimuli with white noise temporal profiles in mice. White noise stimuli are expected to keep the retina in a physiologically more natural state than, e.g., flashes. The influence of mean luminance (ML) was studied.

**Methods:**

Electroretinograms to luminance temporal white noise (TWN) modulation (wnERGs) were measured. The white noise stimuli contained all frequencies up to 20 Hz with equal amplitudes and random phases. Responses were recorded at 7 MLs between −0.7 and 1.2 log cd/m^2^. Impulse response functions (IRFs) were calculated by cross correlating the averaged white noise electroretinogram (wnERG) responses with the stimulus. Amplitudes and latencies of the initial trough and subsequent peak in the IRFs were measured at each ML. Fourier transforms of the IRFs resulted in modulation transfer functions (MTFs). wnERGs were averaged across different animals. They were measured twice and the responses at identical instances in the 1st and 2nd recordings were plotted against each other. The correlation coefficient (*r*^2^_repr_) of the linear regression quantified the reproducibility. The results of the first and second measurement were further averaged. To study the underlying ERG mechanisms, the ERG potentials at the different MLs were plotted against those at the lowest and highest ML. The correlation coefficients (*r*^2^_ML_) were used to quantify their similarities.

**Results:**

The amplitudes of the initial (a-wave-like) trough of the IRFs increased with increasing ML. The following positive (b-wave-like) peak showed a minimum at −0.4 log cd/m^2^ above which there was a positive correlation between amplitude and ML. Their latencies decreased monotonously with increasing ML. In none of the IRFs, oscillatory potential (OP)-like components were observed. *r*^2^_repr_ values were minimal at a ML of −0.1 log cd/m^2^, where the MTFs changed from low-pass to band-pass. *r*^2^_ML_ values increased and decreased with increasing ML when correlated with responses obtained at the highest or the lowest ML, respectively.

**Conclusion:**

White noise electroretinograms can be reliably recorded in mice with luminance stimuli. IRFs resemble flash ERGs superficially, but they offer a novel procedure to study retinal physiology. New components can be described in the IRFs. The wnERGs are either rod- or cone-driven with little overlap.

## Introduction

Electroretinography (ERG) is a valuable tool to study retinal physiology and integrity *in vivo* and non-invasively. It is therefore a very common technique employed in clinical routine and in basic research. The conventional ERG is elicited by flashes. The flash ERG has a characteristic waveform that is well studied and that contains wave components [e.g., a- and b-wave, oscillatory potentials (OPs) and the photopic negative response (PhNR)] with generally known cellular origins (Frishman, 2006). Pathophysiological processes may lead to alterations in these components, thereby providing information about the affected retinal neurons and mechanisms. To ensure comparisons of results from different institutions, the ERG recordings are standardised by the International Society for Clinical Electrophysiology of Vision (ISCEV) (Robson et al., 2022). Although flash ERGs are well studied and standardised, a bright flash is an unnatural and possibly even unphysiological stimulus. For instance, a 10 cd s/m^2^ 5 ms flash would provide about 100,000 td retinal illuminance in a human subject with dilated pupils. Such a stimulus is highly unnatural in a dark-adapted state and may push the retina in an extreme mode of operation where non-linear mechanisms may become apparent that are not active under more natural stimuli (even when the stimulus is not hazardous). We previously proposed that OPs may be the result of such a non-linearity (Zele et al., 2017; Kremers et al., 2022).

In addition to the flash, ERGs are measured to a variety of different stimulus waveforms such as sawtooth and sinewaves (Viswanathan et al., 2004; Pangeni et al., 2010, 2012; Gowrisankaran et al., 2013; McAnany et al., 2013). Most of these stimuli have a simple temporal structure and the analysis of the responses are generally straightforward. Furthermore, not only the temporal but also the spectral and spatial properties of the stimulus can be varied (Porciatti, 2007; Dutescu et al., 2013; Tsai et al., 2015; Joachimsthaler et al., 2017). Stimuli with white noise temporal profiles are more closely to natural stimuli as they have less regular statistics than most stimuli used in experimental procedures. Although used in other types of vision research (Chichilnisky, 2001; Chichilnisky and Rachel, 2002) they were, until recently, rarely employed in ERG recordings (Saul and Still, 2016; Zele et al., 2017; Adhikari et al., 2019; Wang et al., 2019; Kremers et al., 2022). Temporal white noise (TWN) stimuli have several advantages in comparison with flashes. First, in contrast to flashes, the energy is not compressed in a very short time (see above) but spread along the whole recording time, and thus the stimulus keeps the retina in a physiological mode of operation. Second, the response that can be used for analysis is not limited to a time window after a flash (as the response is present during the whole recording time) thereby increasing the signal-to-noise ratio (SNR). Third, long intervals between stimuli that are necessary for flashes (for recovering the adaptation to the background) are not required. Fourth, in contrast to the flash ERG the mean luminance (ML) is independent of stimulus strength and thus can be used as an invariant that can be easily studied. Fifth, the spectral content of the TWN stimuli can also be varied without a change in the state of adaptation. Thus, silent substitutions to isolate the responses of single photopigments are possible. In the present study, luminance stimuli are used. The use of photoreceptor isolating stimuli using the silent substitution technique is the subject of a subsequent investigation.

The purpose of the present study is to present ERG responses to TWN stimuli (wnERGs) at different MLs from the mouse retina and to describe underlying physiological mechanisms. The cross-correlation of the wnERGs with the TWN stimulus is used to extract the impulse response function (IRF). In human subjects and in non-human primates, the impulse response functions (IRFs) resemble standard flash ERGs (Zele et al., 2017; Kremers et al., 2022) with an initial a-wave-like negativity that is followed by a positive deflection similar to the b-wave and by a component that resembles the PhNR. In contrast to the typical flash ERG response, the IRFs showed no oscillatory potential (OP)-like components in both humans and non-human primates (Zele et al., 2017; Kremers et al., 2022). It is assumed that the same cell types contribute to the waveform of the IRF as to the flash ERG. In the present study, we extract the IRFs at different MLs and compare them with flash ERGs in mice. Since the IRF is an approximation of the flash response for a linear system, the comparison will therefore provide insight in which non-linearities are involved in the flash ERG. Finally, a linear regression of the wnERGs to repeated measurements is performed to quantify their reliability. The linear regression of the wnERGs obtained at different luminance levels quantifies the similarity of the responses obtained at different states of adaptation and thus of their underlying retinal mechanisms (e.g., concerning their inputs from rod and/or cone). The Fourier transform of the IRF results in the modulation transfer function (MTF) that describes how signals of different temporal frequencies are processed by the linear approximation. We will discuss the analysis of wnERGs obtained in mice and compare the received IRFs of the luminance modulation and standard flash ERGs and IRFs of mice (Wang et al., 2019), macaques (Kremers et al., 2022), and human subjects (Zele et al., 2017).

## Materials and methods

### Animals

All animal experiments were performed in accordance with the principles regarding the care and use of animals adopted by the Association for Research in Vision and Ophthalmology (ARVO). The conductance of these experiments was approved by the local ethics authorities (Regierungspräsidium Mittelfranken, Ansbach, Germany). For all ERG recordings Opn1lw^LIAIS^ mice on a C57Bl/6J background (hereafter referred to as LIAIS mice) were used. LIAIS mice express the human L-cone pigment instead of the native M-cone pigment. Physiologically they are identical to wild-type mice (Greenwald et al., 2014; Tsai et al., 2015; Joachimsthaler et al., 2017). These mice were used because they also underwent recordings with cone- and rod-isolating stimuli, the results of which are not part of the present study. The LIAIS strain was kindly provided by Profs. Maureen and Jay Neitz from the University of Washington (Seattle, WA, USA) and housed and bred in the Transgenic Mouse Facility in Erlangen, Germany. The mice were kept in a 12 h light/12 h dark cycle with water and food available *ad libitum*.

For the white noise electroretinogram (wnERG) recordings, 11 hemizygous male LIAIS mice at an age between 14 and 20 weeks (mean: 16.35 ± 1.69 weeks of age) were used. Recordings were performed in up to four sessions that were at least 1 week apart. Internal noise signal recordings were performed on additional five mice (13.14 weeks ± 0.35 of age) and control measurements to flash stimuli were done on another group of nine mice (16.75 weeks ± 0.42 of age).

### Preparation

After dark adapting overnight all further handling was performed under dim red light to keep the animals dark adapted (LIAIS mice may be sensitive to this light due to the presence of the L*-pigment; However, pilot studies in our lab have shown that there were no differences between LIAIS and WT animals neither when the preparation was performed under dim red nor under infra-red conditions - Stüwe, Stallwitz, Kremers, and Joachimsthaler, unpublished data). The animals were anaesthetised by a mixture of 50:10 mg/kg ketamine/xylazine (Ketavet; Pfizer, Karlsruhe, Germany; Rompun 2%; Bayer AG, Leverkusen, Germany) that was injected intramuscularly. One drop of tropicamide (Mydriaticum Stulln, 5 mg/ml; Pharma Stulln, Stulln, Germany) and of phenylephrine-hydrochloride (Neo-synephrin POS 5%; Ursapharm, Saarbrücken, Germany) each were applied topically to dilate the pupils of the animals. To prevent dehydration whilst the animals were anesthetised, an injection of 400 μl saline (0.9%) was given prior to recordings. The animals were placed on the heated platform of the Celeris System (Diagnosys LLC, Cambridge, UK) to maintain body temperature during ERG recordings. Needle electrodes, placed subcutaneously at the base of the tail and medially to the ears served as ground and reference, respectively. Two full-field light guide stimulator electrodes (Diagnosys LLC, Cambridge, UK) filled with Corneregel (Dr. Mann Pharma, Berlin, Germany) were placed on the two eyes to serve as active electrodes and full-field stimulators at the same time. The two eyes were stimulated simultaneously.

### White noise electroretinograms

**FIGURE 1:**
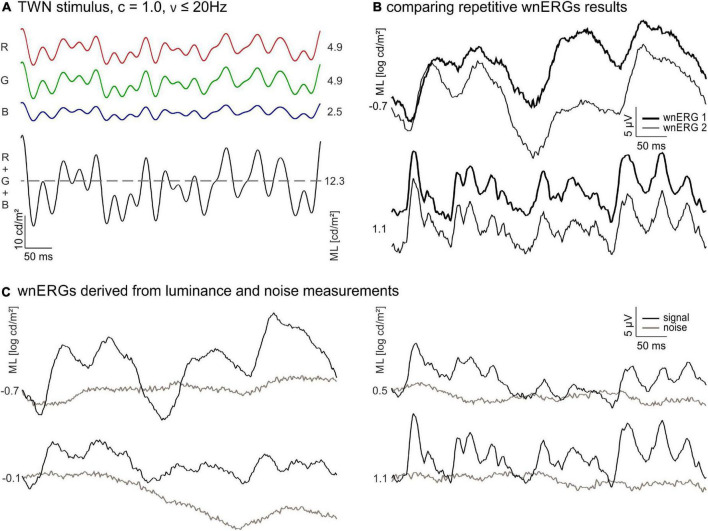
White noise electroretinograms (wnERGs) in mice. **(A)** The temporal white noise (TWN) stimulus is generated by three light emitting diodes (LEDs) [red (R), green (G), and blue (B)]. Their mean luminances (ML) adds up to the ML of the stimulus (black). The Michelson contrast of the stimulus is 100% and no frequencies above 20 Hz were presented. **(B)** Two repeated wnERGs measured at the same ML (thin and thick line) for two different MLs. The responses are averages from all animals. **(C)** Repeated white noise electroretinogram (wnERG) recordings of **(B)** are further averaged for different MLs (black), corresponding noise measurements for each ML are shown in brown.

For the recordings of wnERGs a TWN stimulus was presented. The stimuli were generated by the full-field light guide electrodes of the Celeris equipment, which transmit the light of a red, green, and blue light emitting diode (LED) with emission spectra with peaks (λ_max_) at 630 nm (red; 19 nm full width at half maximum, fwhm), 532 nm (green; 32 nm fwhm), and 452 nm (blue; 19 nm fwhm). Stimulation was controlled by the Espion software (Diagnosys LLC, Cambridge, UK). The luminance of the stimulus was modulated around a mean and had a Gaussian distribution. Stimuli were presented in 512 ms sweeps. The Michelson contrast in a sweep [defined as (L_max_ − L_min_)/(L_max_ + L_min_), where L_max_ and L_min_ are the maximal and minimal luminance in the sweep, respectively] was 100%. Owing to the Gaussian luminance distribution (L_max_ + L_min_) is about twice the ML. The stimulus was created by performing an inverse Fourier transformation of the stimulus in the frequency domain with equal amplitudes of frequencies up to 20 Hz and with random phases at each frequency (see Figure 1; Zele et al., 2017). Frequencies above 20 Hz were not included in the stimulus, because ERG responses to these frequencies are very small in mice (Tsai et al., 2015, 2017) and therefore barely contribute to the wnERG. Furthermore, they resembled natural noise stimuli, that were used previously (Saul and Still, 2016; Wang et al., 2019), more closely. In the present study, the TWN stimuli were luminance modulations (i.e., all LEDs were modulated simultaneously with equal contrast and time course). The form of the TWN stimuli was identical for all measurements; only the ML was altered. Luminance modulated TWN stimuli exhibited a R:G:B ratio of 2:2:1 which resulted in a slightly purplish colour. This ratio was used because the recordings were repeated with stimuli that isolated the responses of single photopigment types (results of these recordings are not presented here). With the mentioned ratio, the outputs of the different photopigments (in terms of rod- or cone-contrast) were optimised. The stimuli were presented at −0.7, −0.4, −0.1, 0.2, 0.5, 0.8, and 1.1 phot log cd/m^2^ (i.e., 0.19, 0.38, 0.77, 1.53, 3.1, 6.1, and 12.3 phot cd/m^2^; equal to 0.89, 1.77, 3.59, 7.13, 14.45, 28.43, and 57.32 scot cd/m^2^) ML. Prior to the first recording, the animal was adapted for 1 min to a background light with the ML of the following stimulus. At each ML, two recordings to 200 sweeps each (512 ms per sweep; the luminance was refreshed every ms and the rate therefore was 1 KHz) were performed. In between sweeps and MLs there was no interstimulus interval. The recording to the first sweep was discarded to avoid onset artefacts. The two recordings were used to obtain an estimate of the reproducibility of the recording. In further analyses, the responses to the two recordings were averaged. The recordings for all seven MLs were done in one recording session. A recording session lasted around 50 min after which the animals were allowed to wake up.

### Internal noise

We also performed measurements to a steady background at the same ML as the wnERG recordings to be able to estimate internal noise in the ERG signal. These measurements were performed on a different group of LIAIS mice and at every ML used during the wnERG recordings after 5 min of adaptation to the first shown ML. Again, the internal noise measurements were performed twice with 200 sweeps per measurement. The protocol lasted around 50 min and the animals were allowed to wake up afterward.

### Flash electroretinograms

For comparison with the IRFs, obtained from the wnERGs (see below), flash ERGs were measured using the Diagnosys Celeris System. Similar to the TWN stimuli, the flashes were created by the red, green, and blue LEDs with a ratio of 2:2:1. Mice were adapted to a steady background (BG) luminance of either −0.6 or 1.2 log cd/m^2^ (i.e., 0.225 and 14.4 cd/m^2^). Flashes of −1.0 and 0.8 log cd s/m^2^ (i.e., 0.1 and 6.5 cd s/m^2^) were displayed on the −0.6 and 1.2 log cd/m^2^ BGs (see Table 1), respectively, resulting in Weber fractions of about 112. The inter-stimulus interval was 10.5 s with a −0.6 log cd/m^2^ BG and 0.5 s with 1.2 log cd/m^2^ BG. The flash duration was 4 ms. For −0.6 and 1.2 log cd/m^2^ BGs, the responses to 10 and 30 flashes were averaged, respectively.

**TABLE 1:** Control flash electroretinogram (ERG) stimuli settings.

	Total BG 0.225 cd/m^2^	Flash 0.1 cd s/m^2^, 4 ms	Total BG 14.4 cd/m^2^	Flash 6.5 cd s/m^2^, 4 ms
Red LED	0.09 cd/m^2^	10.09 cd/m^2^	5.76 cd/m^2^	645.76 cd/m^2^
Green LED	0.09 cd/m^2^	10.09 cd/m^2^	5.76 cd/m^2^	645.76 cd/m^2^
Blue LED	0.045 cd/m^2^	5.045 cd/m^2^	2.88 cd/m^2^	322.88 cd/m^2^

White noise electroretinogram, internal noise and flash ERG recordings were band-pass filtered between 0.125 and 300 Hz. The signal was amplified eight times and the sampling frequency was 1,000 Hz.

### Data analysis

#### Impulse response functions

**FIGURE 2:**
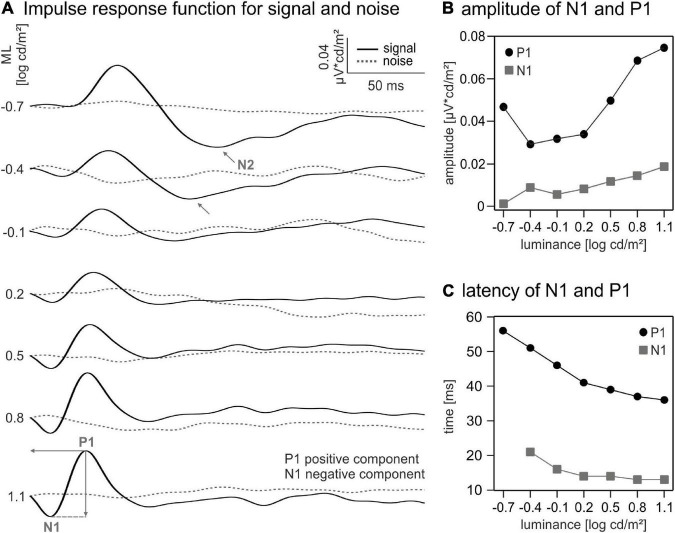
Impulse response functions (IRFs) and curve components. (A) IRFs for different mean luminances (MLs) calculated using white noise electroretinogram (wnERG) signals (black) and noise measurements (dotted line). Indicated wave components: N1 for first negative trough, P1 for first positive peak, and N2 for second negative trough. (B) Amplitudes and (C) latencies of P1 (black dots) and N1 (grey squares).

To obtain the IRFs, the averaged wnERG for each luminance was cross-correlated with the TWN stimulus: The wnERG potential was multiplied with the luminance of the TWN stimulus at each instant and summed for all 512 timestamps (Zele et al., 2017). The procedure was repeated after shifting the wnERG relative to the TWN stimulus in 1 ms steps 256 times. The sum of multiplication plotted as a function of the time shift resulted in the IRF. The cross-correlations were normalised to the ML of the TWN stimulus to obtain IRFs that were independent of the ML. The resultant IRFs are given in Figure 2. Two prominent IRF wave components (i.e., an initial negative–N1- and a subsequent positive–P1-component; see Figure 2) could be identified for all MLs, at lower MLs a second negative deflection (N2) was visible.

The amplitude of the N1 component was defined as the difference between the baseline (defined as the mean of the first 6 ms in the IRF) and the N1 trough (within a time window between 10 and 30 ms). The amplitude of the positive component was measured from the N1 trough to the P1 peak (within a time window between 30 and 60 ms). The latencies of the N1 and P1 were defined as the time shifts at their minimum (N1) or maximum (P1).

Similarly, the amplitude of the N2 component was defined as the difference between the baseline (see above) and the N2 trough. N2 latency was defined as the time shift at its minimum.

#### Reproducibility

The responses to the TWN stimuli in the first recordings of 200 sweeps (wnERG) were averaged for all eyes and all animals. Similarly, the responses in all second recordings were averaged. Subsequently, the two averaged potentials at each time point during the recording were plotted against each other. A linear regression through these data was used to obtain the correlation coefficient *r*^2^_repr_, which quantified the reproducibility of the wnERGs. The correlation coefficient varied between 0 (indicating the absence of any similarity between the two recordings) and 1 (meaning complete accordance of both results).

Subsequently, the two repeated recordings one and two were further averaged and the potentials of each wnERG were plotted against those obtained at the highest ML, to see how well the wnERGs recorded with different MLs coincide. Similarly, they were plotted against those obtained at the lowest ML. Again, correlation coefficients (*r*^2^_ML_) were obtained to quantify the similarities of the responses at intermediate MLs with those obtained at the highest and lowest ML.

#### Modulation transfer function

By performing a fast Fourier transformation (FFT) of the IRFs the so-called MTF was obtained. The FFT converts the IRFs into a representation in the frequency domain where the amplitudes and phases are given as a function of temporal frequency. For a linear response system, the MTF is identical to the response amplitudes and phases to sinewaves at the given temporal frequency.

## Results

### White noise electroretinograms at different mean luminances

Figure 1A displays the TWN stimulus. The wnERGs (averaged across animals) from the 1st and 2nd recordings at −0.7 and 1.1 log cd/m^2^ MLs are shown in Figure 1B. Obviously, the two recordings at the same ML have similar morphologies, while the recordings at two different MLs differ substantially. This indicates that wnERGs are reproducible at the same ML, but the underlying mechanisms at the two MLs are different.

The grand averages (i.e., the additional average of the first and second recordings) of recordings at four different MLs from low mesopic (−0.7 log cd/m^2^) to photopic (1.1 log cd/m^2^) are shown in Figure 1C. Averaged internal noise measurements at each ML are also displayed. Again, it can be seen that the recordings at −0.7 and at 1.1 log cd/m^2^ ML differ substantially, indicating that different retinal mechanisms are responsible for the measured ERGs. The wnERGs at 1.1 log cd/m^2^ contain faster modulations. Furthermore, the responses at the other MLs display intermediate characteristics, suggesting a transition of underlying mechanisms.

### Flash electroretinograms and impulse response functions

We derived IRFs by cross-correlating the wnERGs with the TWN stimuli (see “Materials and methods” section). IRFs obtained at the different MLs are displayed in Figure 2A. They were remotely similar to standard flash ERGs. An initial (a-wave-like) negative deflection (N1) was followed by a positive (b-wave-like) peak (P1), and resembled IRFs obtained by other groups in human subject (Saul and Still, 2016; Zele et al., 2017; Adhikari et al., 2019), mice (Wang et al., 2019), and macaques (Kremers et al., 2022). OP-like components were not present. The P1 component is clearly discernible even at the lowest ML, while N1 is at noise level at the lowest ML (−0.7 log cd/m^2^). The amplitudes of N1 increased with increasing ML, whereas the P1 showed a minimum at a ML of about −0.4 log cd s/m^2^. The latencies of both components decreased with increasing ML. The IRFs at −0.7 and −0.4 log cd/m^2^ showed a second negative deflection (N2) following P1. N2 amplitudes and latencies decreased from 0.0462 μV cd/m^2^ at 115 ms to 0.0344 μV cd/m^2^ at 103 ms. The component vanished for higher MLs.

The quantitative analysis of the amplitudes and latencies of the IRF components (Figure 2B) revealed a non-monotonic relationship between the amplitude of P1 and ML. An initial 1.6-fold decrease between −0.7 and −0.4 log cd/m^2^ ML is followed by a 2.56-fold increase in amplitude from −0.4 to 1.1 log cd/m^2^ ML. In contrast, the amplitude of N1 shows a monotonic (15.28-fold) increase between −0.7 and 1.1 log cd/m^2^ ML. The latencies of N1 and P1 decreased monotonously by about 10 and 20 ms, respectively, from the lowest to the highest ML (Figure 2C).

We also cross-correlated the wnERG of the internal noise measurements with the TWN stimuli. The resulting waveforms did not show any systematic response (Figure 2A dashed lines) proving that the wnERGs measured with the TWN stimulus indeed contained a significant signal and that the resulting IRFs had a physiological origin.

**FIGURE 3:**
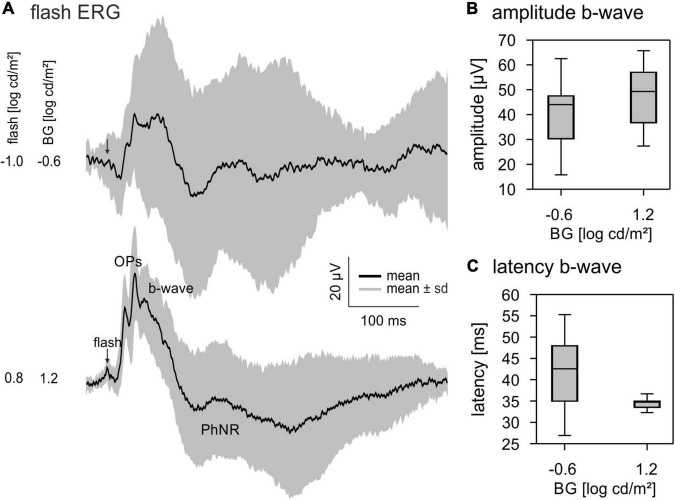
Flash electroretinograms (ERGs). (A) Averaged ERG responses to a –1.0 log cd s/m^2^ flash on a –0.6 log cd/m^2^ BG and a 0.8 log cd s/m^2^ flash on a 1.2 log cd/m^2^ BG. Black lines show the averaged signals, the grey area represents mean ± standard deviation. (B) Quantitative analysis of the b-wave amplitude and (C) latency. Boxplots show median and 25 and 75% quartiles. Whiskers represent the minimal and maximal values of the dataset.

We measured flash ERGs for comparison with the IRFs. ERGs were measured to 4 ms flashes with a −1.0 log cd s/m^2^ flash on a −0.6 log cd/m^2^ background and a 0.8 log cd s/m^2^ flash on a 1.2 log cd/m^2^ background. The ERGs had a small initial a-wave followed by a positive b-wave (Figure 3A). OPs were visible for both luminance conditions; however, they were more distinct at 1.2 log cd/m^2^ flash strength. In addition, a PhNR was visible at about 150 ms post-flash. The quantitative analysis of flash ERGs showed a larger and faster b-wave for the higher luminance condition (Figures 3B,C).

### Correlation coefficients

As shown above, the wnERG waveforms were quite different for different MLs (Figure 1C), while the recordings were reproducible when measurements were repeated at the same ML (Figure 1B). We therefore propose that different retinal pathways were responsible for the wnERGs at different MLs.

**FIGURE 4:**
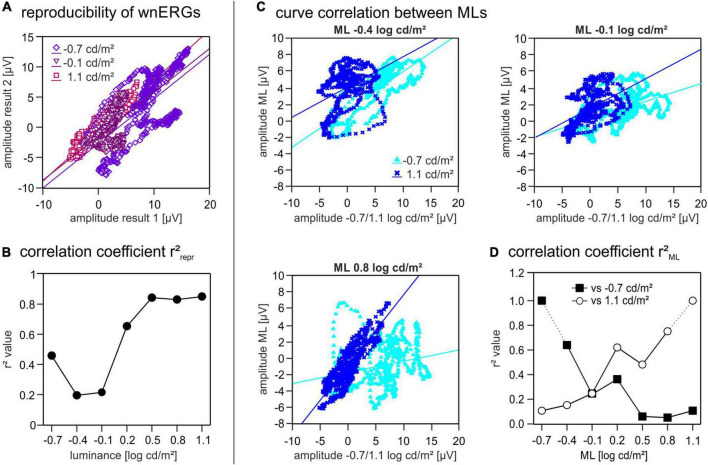
Correlation coefficients. (A) Two repeated white noise electroretinogram (wnERG) recordings at one ML are plotted against each other [for three different mean luminances (MLs)]. Amplitudes of the first recording are shown on the abscissa and amplitudes of the second recordings are shown on the ordinate. Linear regression lines were fitted through all data sets. (B) *r*^2^_repr_ values are plotted as a function of ML. (C) First and second wnERG recordings were further averaged at each ML. To study curve correlation between different MLs the wnERGs at all MLs were plotted against those obtained at –0.7 and at 1.1 log cd/m^2^ on the *X*-axis. Potentials obtained at three different ML (–0.4, –0.1, and 0.8 log cd/m^2^) are shown on the *Y*-axis. Linear regression lines were fitted through all data sets. (D) Linear regression of C gave two correlation coefficients *r*^2^_ML_ for each ML (filled squares when plotted against –0.7 log cd/m^2^, open circles when plotted against 1.1 log cd/m^2^).

To further investigate these findings, we first quantified the reproducibility of the wnERG recordings at each luminance by plotting the potentials measured at equal instances during the 1st and 2nd recordings against each other and by calculating the correlation coefficients (*r*^2^_repr_) of the linear regressions (Figure 4A). The plot in Figure 4B shows the resulting *r*^2^_repr_ values in dependence of the ML. This plot clearly displays a minimum for the *r*^2^_repr_ values around −0.4 log cd/m^2^ ML (Figure 4B) indicating a weak correlation between the two recordings and therefore a low reproducibility at MLs −0.4 and −0.1 log cd/m^2^. At −0.7 log cd/m^2^ ML the *r*^2^_repr_ value is around 0.5 and therefore reflects an intermediate reproducibility of the recordings. At higher MLs the *r*^2^_repr_ values increase up to 0.8, therefore showing a stronger correlation and good reproducibility of the recordings.

To see, how well the response characteristics were preserved at different MLs, we plotted the mean wnERG potentials (i.e., after further averaging the responses obtained from the 1st and 2nd measurements) for each luminance against those obtained at equal time points during the recordings at −0.7 log cd/m^2^ ML. Furthermore, the potentials at each luminance were plotted against the values obtained at 1.1 log cd/m^2^ ML (Figure 4C shows three examples of plots). To assess the resemblance of the signals at the different MLs relative to each other, we calculated the correlation coefficients (*r*^2^_ML_) of the linear regressions (Figure 4D). The comparison with the responses obtained at −0.7 log cd/m^2^ ML (Figure 4D, black squares) revealed *r*^2^_ML_ values that decrease with increasing ML, whereas those of the correlation with the data obtained at 1.1 log cd/m^2^ ML increased with increasing ML (Figure 4D, open circles). The two plots cross at a value of ML of −0.1 log cd/m^2^, thus coinciding with the luminance region where *r*^2^_repr_ was minimal. This cross point also divides the luminance range in two separate luminance ranges. wnERGs showed a high correlation when compared to recordings within the same luminance range, whereas there was poor correlation when compared to recordings of the other luminance range.

### Modulation transfer function

The modulation transfer functions (MTFs) were obtained by performing an FFT on the IRFs. MTFs show the amplitudes and phases of the signal as a function of the temporal frequency.

**FIGURE 5:**
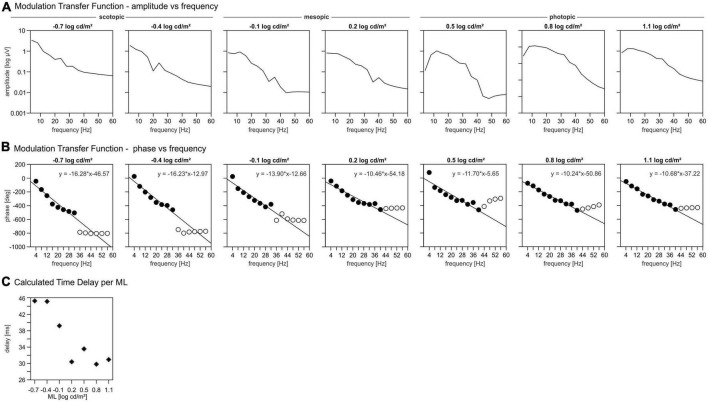
Modulation transfer functions (MTFs). (A) Amplitudes of the signal per mean luminance (ML) as function of the temporal frequency. (B) Phases of the signal per ML as function of the temporal frequency. Closed symbols: phases included in further analysis for delay, open symbols: phases excluded for further delay analysis. (C) Calculated time delay per ML.

The plots displaying the MTF amplitudes vs. temporal frequency in Figure 5A show three different groups of MTF characteristics that divide the total luminance range in a scotopic, mesopic, and photopic ML range. The amplitudes at the lower ML range from −0.7 to −0.4 log cd/m^2^ decrease monotonously with increasing frequency (Figure 5A). At intermediate MLs (between −0.1 and 0.2 log cd/m^2^) the amplitudes were constant between 3 and 13 Hz. At higher frequencies, the amplitudes decrease continuously with increasing frequency. At MLs between 0.5 and 1.1 log cd/m^2^, the MTFs show a maximum at about 10 Hz above which the amplitudes decrease again (Figure 5A).

The MTF phases are plotted vs. temporal frequency in Figure 5B up to 58 Hz. As temporal frequency increases, the phase decreases approximately linearly up to a frequency of 32 Hz (MLs < 0.2 log cd/m^2^) or 40 Hz (MLs > 0.2 log cd/m^2^), above which the phases are fairly constant for all MLs. However, the amplitudes at these high frequencies were too small to obtain reliable phases and thus were disregarded in further analysis. A linear relationship between phase and frequencies indicates that the responses are probably determined by a fixed time delay. The slope of the linear regression through the phase vs. frequency data quantifies the apparent delay time. These linear regressions (in deg/Hz) are given in Figure 5B. The delays can then be calculated from the slopes of the linear regressions by dividing the slopes by 360°. The apparent delays are displayed as a function of ML in Figure 5C. The delays vs. ML data follow a sigmoid function, that can be divided in three parts; for MLs lower than −0.1 log cd/m^2^ the delays are largest with values around 45 ms. For MLs equal or larger than 0.2 log cd/m^2^ the delay values are minimal and about 30 ms. The delay at −0.1 log cd/m^2^ is 39 ms and thus intermediate.

## Discussion

### Comparison between primate and mouse impulse response functions and flash electroretinograms

In this study wnERGs in mice were measured using a TWN stimulus. The cross correlation between the two results in the IRF. IRFs were previously obtained for human observers (Saul and Still, 2016; Zele et al., 2017; Adhikari et al., 2019), mice (Wang et al., 2019), and macaques (Kremers et al., 2022). All IRFs have a characteristic morphology with an initial negative deflection (N1) that is followed by a positive peak (P1). These resemble the a- and b-waves of flash ERGs. Since IRFs and flash responses are identical in linear systems (Zele et al., 2017), we suggest that the N1 component is homologue to the a-wave. Similarly, the P1 and the b-wave are probably homologues. In agreement with this proposal, the latencies of N1 and P1 decrease with increasing ML in mice (Figure 2B) and human subjects (Zele et al., 2017), comparable to those of the a- and b-waves of the flash ERG (Figure 3; Herrmann et al., 2010): P1 latency decreases from 56 to 35 ms between −0.7 and 1.1 log cd/m^2^ ML, whereas b-wave latencies decrease from 43 to 35 ms in the same background luminance range. Furthermore, the amplitudes of N1 (Figure 2B) and the a-wave of flash ERGs increase with increasing ML (Herrmann et al., 2010). This was also observed for human IRF N1 components (Zele et al., 2017) and for human flash ERGs (Frishman et al., 1996). However, whereas the b-wave amplitude increases continuously with increasing ML in mice (Herrmann et al., 2010), the P1 amplitudes in this study decrease with increasing ML for MLs up to −0.4 log cd/m^2^ above which they increase again. The IRF data suggest a transition from rod to cone driven responses (see also below). We propose that the increase in amplitude and decrease in latency of N1 and P1 above −0.4 log cd/m^2^ can be attributed to an increasing contribution of cone driven signals (Figure 2).

N1 latencies are similar (about 15 ms) for mice, humans and macaques; the latency of P1 is, however, larger in mice (35 ms in mice vs. 24 ms in human observers and 20 ms in macaques). Zele et al. (2017) also showed a reduction in latencies with an accompanying increase in the amplitudes of N1 and P1 with increasing ML.

Interestingly, we found that the mouse IRFs at low MLs show a second negativity (the N2) following the P1 component. The amplitude of this N2 component is maximal at the lowest ML and both, amplitude and latency of N2, decrease with increasing ML (although this finding remains to be confirmed because the component was found at only two MLs). The absence of this component at mesopic and photopic ML conditions suggests a mostly rod driven origin. The N2 component is possibly similar to the scotopic threshold response (STR), which is a late and negative wave in the flash ERG elicited by very weak flashes at dark adaptation (Saszik et al., 2002). Saszik et al. (2002) found a peak time of the STR of around 200 ms and about 100 ms after the peak of the b-wave. The N2 of the IRFs have a latency of about 125 ms and appear about 75 ms after the peak of P1. Nevertheless, its characteristics, a late and negative curve progression decreasing in amplitude with increasing luminance and being absent at higher luminances, may point at commonalities. We therefore propose that the N2 component in the IRF is homologue to the STR in the flash ERG and therefore may reflect activity of the retinal ganglion cells. However, the N2 component is fairly easy to obtain with good signal-to-noise ratio, whereas the STR is often difficult to measure because very weak flashes have to be employed. This proposition remains to be studied, possibly by using intravitreal injections of gamma aminobutyric acid (GABA), which has shown to abolish the STR (Saszik et al., 2002).

Impulse response functions (IRFs) of primates also display late deflections that are, however, positive. Furthermore, whereas the late component occurs at low luminances in mice, they are most prominent at high luminances in macaques (Kremers et al., 2022) and human observers (Zele et al., 2017). The late components in primates have been proposed to be a possible correlate of L-M-cone opponency in the parvocellular pathway (Kremers et al., 2022). This pathway is absent in the mouse, thereby possibly explaining why it cannot be found in mouse IRFs.

Similar to IRFs in human observers (Zele et al., 2017) and macaques (Kremers et al., 2022) OPs are not present in mouse IRFs. However, they are generally very prominent in mouse flash ERGs (see Figure 3; Harazny et al., 2009). As suggested previously for human observers (Zele et al., 2017) and macaques (Kremers et al., 2022) we propose that the OPs are the result of non-linearities in the flash ERGs due to the high retinal illuminance during the flash stimulation. The Weber contrast in flashes are orders of magnitude larger than those contrasts in TWN stimuli. It should be mentioned, however, that contrasts in the continuous TWN stimuli are probably more adequately described by Michelson contrast rather than Weber contrast which is more appropriate for describing the strength of short flashes (Kremers et al., 2005). Although OPs are prominent in mouse flash ERGs, it must be noted that they are very small for low flash strengths and strongly increase with increasing flash strength (Harazny et al., 2009; Falk et al., 2019). The high retinal illuminance during short flashes is possibly non-physiological resulting in large non-linearities. As with natural stimuli, the luminance contrasts of the TWN stimulus are much smaller, possibly resulting in the absence of OPs.

### Transition between rod and cone driven responses

Our data suggest a sharp transition between rod and cone driven responses at a ML of about −0.1 log cd/m^2^. This conclusion is based on five observations: (i) The wnERGs show a clear change in waveforms (Figure 1), which is substantiated by the fact that at lower MLs, the responses are more strongly correlated with each other but not with those at higher MLs and *vice versa* (Figure 4D). (ii) The IRFs display a transition at this ML, where the P1 component is minimal (Figure 2B) and the latencies of the N1 and P1 components display a change in dependency on ML (Figure 2C). (iii) A N2 component is only present at lower MLs. (iv) The MTFs also display a transition in the frequency domain at this ML (Figure 5), which is in agreement with earlier data on mouse ERGs elicited by sinusoidal stimuli of different temporal frequencies (Tsai et al., 2015, 2017). This transition in the frequency dependent responses is accompanied by a shift in apparent delay from 46 ms at low MLs (similar to the delay of 40–53 ms in rod driven responses; Tsai et al., 2017) – to about 32 ms at high ML (which again is similar to the 33–37 ms delay found previously in cone driven responses; Tsai et al., 2017). The transition ML coincides with the ML where P1 of the IRFs is minimal. (v) The reproducibility of the wnERG is minimal at the ML of transition (Figure 4B), indicating that the responses are poor at this transition ML. This would suggest that rod and cone driven signals have clearly separated and non-overlapping ranges of activities and that a mesopic range, as is found in primates, is absent or much narrower in mice. This clear separation of rod and cone driven signals was also shown in a previous study of our group using sinewave stimuli (Tsai et al., 2017), where rods gave robust signals up to 1.4 cd/m^2^, whereas cones showed reliable signals at 7 cd/m^2^ and higher but with weaker response to both rod and cone isolating stimuli at intermediate MLs. The current study narrows this intermediate range to about −0.1 log cd/m^2^ (i.e., 0.77 cd/m^2^).

### Further developments

With the use of TWN stimuli, which displays characteristics that are closely related to those of natural scenes, Saul and Still (2016) and Wang et al. (2019) could further improve their measurements with regard to comfort for the patients and increased robustness of obtained responses.

In contrast to flashes, the TWN stimuli can be easily combined with the silent substitution stimulation technique to isolate the responses of single photoreceptor types. In a subsequent study, we will use the silent substitution technique in combination with TWN stimuli for the first time in mice to study rod- and cone-driven wnERGs, IRFs, and MTFs separately.

## Conclusion

The TWN stimulus can be used to elicit ERGs (wnERGs) that can characterise the properties of ERG generating mechanisms in a very efficient manner. IRFs can be used to describe components similar as in the flash-ERG but in the absence of non-linearities. MTFs provide information that resemble those obtained with ERG responses to sinusoidal modulations at a multitude of temporal frequencies.

In addition, the comparison of wnERGs obtained in different animal species (here particularly primates) can be used to describe basic differences and similarities in the retinal physiology.

Finally, the correlation between wnERGs obtained at different adaptation conditions gives information about the similarity and difference between underlying mechanisms and about ranges of transition between these mechanisms. Our data indicate that a mesopic range, where rods and cones are active and directly influencing ERG responses, is absent or at least strongly diminished in mice.

## Data availability statement

The raw data supporting the conclusions of this article will be made available by the authors, without undue reservation.

## Ethics statement

This animal study was reviewed and approved by the Regierungspräsidium Mittelfranken, Ansbach, Germany.

## Author contributions

NS: planning and performance of the experiments, data analysis, interpretation of the results, writing, and editing the manuscript. AJ: conception and design of the study, planning of the experiments, data analysis, interpretation of the results, and editing the manuscript. JK: conception and design of the study, construction of the stimuli, data analysis, interpretation of the results, writing, and editing the manuscript. All authors contributed to the article and approved the submitted version.
